# The Institutional Worker

**Published:** 1918-04-27

**Authors:** 


THE
### INSTITUTIONAL WORKER
**Being a Special Supplement to "The Hospital"**

### OUR BUREAU OF INFORMATION.

### Rules for Correspondents.

1. Every letter must be accompanied by the coupon to be cut from the back cover (inside page) of The Hospital, current issue, and must contain the name and address of the correspondent with pseudonym for publication if desired. Replies by post cannot be given save under exceptional circumstances at the Editor's discretion.

2. Letters from Approved Homes in reply to special needs published in the Bureau should state terms and full particulars, and be sent prepaid under cover to the Editor of the Bureau with name written across coupon for identification.

3. Proprietors of Homes which have not yet been entered on the List of Approved Homes, but have spare accommodation likely to suit special needs, are invited to write for an application form for registration. The fee for registration, which includes two announcements of the Home in the Bureau and other privileges, is 10s.

4. All communications to be addressed to the Editor of The Hospital, 28 Southampton Street, Strand, London, W.C. 2, and marked "Bureau of Information."

### INSTITUTION FACTS AND FIGURES.

### The Admission of Patients.

**Answer to March Question.**

**By F. S.**

**The question was:**

**Is it an advantage to admit patients to small hospitals (under 50 beds) by subscribers' letters, or is the system of admission on a doctor's order only preferable?**

The question of the use of subscribers' letters is one which has been the subject of much debate in recent years and is peculiar to the voluntary system. The matter can be considered from two aspects—(*a*) medical and (*b*) financial.

It is obvious that if an institution is to be of greatest benefit to the general community the patient's condition must be the primary consideration in deciding whether he (or she) should be admitted to the hospital; not his abilities for obtaining subscribers' letters. On the other hand, the financial point of view cannot be ignored. Where a number of institutions are situated in one area and a certain amount of "overlapping" occurs the issue of "letters," especially in connection with workpeople's contributions, will result in a fairer division of the contributions in proportion to the demand for "letters" of the institutions concerned. Moreover, many subscribers greatly appreciate the privilege of being able to recommend patients for treatment, and this point must be remembered when dealing with this matter. If the hospital is an isolated institution, it would appear that no advantage is likely to be gained by the issue of letters.

Another point worthy of consideration is the "waiting list" of patients. Should the institution have sufficient accommodation for its needs, the hardship sometimes placed upon patients by their being required to produce "letters" would be almost absent. It is taken for granted that, whatever method is in force, no patient requiring urgent treatment would be refused admission on the grounds of being unable to produce the necessary letters at the time.

It is difficult to answer this question without a full knowledge of the local conditions applying to the institution concerned, but the general principles mentioned herein may help in the decision.

### Repairs and Renewals.

**Answer to March Question.**

**By H. L.**

**The question was:**

**Describe the system adopted at your institution for dealing with repairs, including (a) the arrangement in force for the periodical examination of the structure, and (b) the method of collecting, recording, and disposing of repair requisitions.**

Repair requisitions are made upon a form of which the following is a copy :—

REQUISITION FOR REPAIRS.

This form must be filled up and sent to the Secretary's Office IMMEDIATELY any Leakage, Stoppage, or other defect, etc., is discovered in or about the premises. Great waste of water arises if taps are out of order.

Nature of Defect............................
..............................................
..............................................
..............................................
Name of Room, Ward, or Place where Defect exists...............................

Date and hour discovered ............191...
A.M. P.M.
Time.......... ........

Date and hour reported...............191...
A.M. P.M.
Time.......... ........

Signature of Person reporting same
..............................................

SECRETARY'S REMARKS.

..............................................
..............................................
..............................................

When Repaired.........day of.........191...
By whom.......................................
Total time occupied, as separate sheet
..............................................

Upon the receipt of this form the secretary and the handyman examine the defect, and it is then determined what shall be done and who is to execute the work of repair, the progress of which is periodically noted. The details required at the foot of the form are subsequently filled in, and the requisitions filed according to the work or department. A separate record of the time occupied in performing the work is made. A periodic inspection of the building is made by the secretary and the handyman, and all structural defects noted on one of the aforementioned forms. An examination is made at least annually of all roofs.

### Text-Books on Heart Disease.

Write to the Manager, The Scientific Press, Ltd., 28 and 29 Southampton Street, Strand, W.C. 2, for a list of these publications. You do not say whether you are a student of medicine or a qualified practitioner. In case you are not, we would warn you against the danger of reading such a text-book with the object of carrying out treatment.—L. A.

### APRIL QUESTION.

**A Register of Nurses' Ward Work.**

How should a book be ruled to give at a glance a record of nurses' different duties in male, female, and children's wards, and time spent at classes, etc.? The hospital is a training-school, and nurses take day and night duty. There are three floors for male patients, two for female patients, and a children's block.

### RULES.

The following rules must be observed:—

1. Contributions must be written on one side of the paper. Brevity and terseness are desirable features. The MSS. must bear the name and address of the sender and be accompanied by coupon to be cut from the back cover (inside page) of the current issue of THE HOSPITAL. A pseudonym must be chosen if the name is not to be published.

2. Contributions must be addressed to the Editor of THE HOSPITAL, Institutional Worker Supplement, 28 & 29 Southampton Street, Strand, London, W.C. 2, should reach him before the end of the current month, and be marked in the left-hand corner "Facts and Figures."

**A minimum payment of Five Shillings will be made for each published answer.**

### ENQUIRIES AND ANSWERS.

### *EMPLOYMENT AND TRAINING.*

**State Aid for Medical Students.**

THE State makes no provision for cases such as yours. There are, however, numerous scholarships in connection with the medical schools attached to the great hospitals of London as well as in connection with the Faculties of Medicine of the universities of the country. Write to the secretaries of the medical schools in London, as, for instance, those in connection with Guy's, the London, St. Thomas's, Middlesex, University College, and King's College Hospitals. Consider what each offers, and, having arrived at a decision, work hard for the object in view. The first step is to pass the preliminary examination. Have you matriculated at London University? Write again and let us know whether we can give you further advice.—PATH. LAB. (aged seventeen).

**Municipal Nursing and Infant Welfare.**

YOUR deafness is undoubtedly a severe handicap, but as you can still carry on a conversation by ear and have learnt the art of lip-reading, there seems to be no reason why you should not employ your nursing ability in some useful sphere. Have you tried to obtain a position such as home sister, where your knowledge of administrative work could be made of use? You should obtain one or more of the leading nursing papers and watch the advertisements for school nurses, etc. Experienced women with nursing training are required for infant welfare work. Write to the Secretary of the Association of Infant Consultations and Schools for Mothers, 4 Tavistock Square, London, W.C.—M. E. H.

### *THE SICK AND IN NEED.*

**Home for Confirmed Invalids.**

THERE are many different homes for confirmed invalids or incurables, with varying terms of admission. You do not say whether you require one for a man, woman, or child, nor do you give such other particulars as are needful in suggesting a suitable home. We give here the names of several homes, and upon hearing from you again we should be pleased to advise you further:—Home for Confirmed Invalids, 36 Aubert Park, Highbury, for invalid ladies of limited means; payment not less than 15s. a week. St. Elizabeth's Home for Incurable Women, 59 Mortimer Street, W.; terms from £20 to £30 per annum. The Royal Hospital for Incurables, West Hill, Putney Heath, S.W. All payments are subject to a possible increase under war conditions. You have omitted to give your address. A coupon should be sent with each inquiry.—E. A.

### MEDICAL AND ADMINISTRATIVE APPOINTMENTS.

### SCALE OF CHARGES FOR MEDICAL APPOINTMENTS, VACANCIES, &c.

VACANT APPOINTMENTS.—Eight Lines and under (an average Line contains Ten Words), 4s.; for every additional Line or part of a Line, 6d.

To THOSE WANTING APPOINTMENTS.—First Twenty-eight Words and under, 2s.; for every Six or part of Six Words beyond, 6d.

To ensure insertion advertisements should reach us by noon on Wednesdays. Telegraphic address: "Ospedale, London." Telephone No.: 2734 Gerrard.

Remittances should be made by cheque or postal order. All advertisements should be addressed to THE MANAGER, "The Hospital," "The Hospital" Building, 28 & 29 Southampton Street, Strand, London, W.C. 2.

### Appointments Vacant.

CHELTENHAM GENERAL HOSPITAL.

The Board of Management invite applications for the post of SECRETARY.

Commencing salary £200 per annum.

Applications, with copies of not more than three recent testimonials, with clerical experience, should be sent to the undersigned, at the Hospital, on or before SATURDAY, May 4th next, from whom particulars of the appointment may be obtained.

By Order,
CLARENCE JAMES, Acting Secretary.
(5122)

COUNTY BOROUGH OF WEST HAM.

TEMPORARY TUBERCULOSIS OFFICER.

The Corporation of West Ham invite applications for the temporary appointment of MEDICAL OFFICER at the Tuberculosis Dispensary at a salary of £500 per annum.

The person appointed must be a registered Medical Practitioner, over 25 years of age, and must devote the whole time to duties under the Corporation. Preference will be given to candidates who have held a House appointment for at least six months in a General Hospital, and who have had special practical experience in the treatment of Tuberculosis in a Sanatorium or otherwise. The possession of a Diploma in Public Health and practical experience in Bacteriological Laboratory work will be deemed additional qualifications for the post.

The officer appointed will be attached to the Public Health Department, and will be required to work under the administrative control of the Medical Officer of Health.

Forms of application can be obtained from, and must be returned to me, with copies of three recent testimonials, not later than noon on SATURDAY, 4th May, 1918.

Canvassing will disqualify.

GEORGE E. HILLEARY, Town Clerk.

Town Hall, West Ham,
18th April, 1918. (5078X)

QUEEN MARY'S HOSPITAL FOR THE EAST END, STRATFORD (179 Beds).

WANTED, HOUSE SURGEON.

Applications must be addressed to the undersigned. Male applicants must be ineligible for Military Service.

A. W. SCRIVENER, Secretary.
(5102)

### Manager's Notices.

**RATES OF SUBSCRIPTION (payable in advance).**

| | | |
|---|---|---|
| UNITED KINGDOM. | | |
| Three Months | (including Postage) ... | 2s. 0d. |
| Six Months | do. ... | 4s. 0d. |
| Twelve Months | do. ... | 6s. 6d. |
| FOREIGN AND COLONIAL. | | |
| Three Months | (including Postage) ... | 2s. 6d. |
| Six Months | do. ... | 5s. 0d. |
| Twelve Months | do. ... | 8s. 8d. |

**All letters on business matters must be addressed to The Manager, "The Hospital," 28 and 29 Southampton Street, London, W.C. 2.**

**Special Rates** will be quoted to those institutions that agree to send all their public notices to THE HOSPITAL, so as to make it a file of such announcements for ready reference.

